# Hippocampal neurogenesis and *Arc* expression are enhanced in high-fat fed prepubertal female pigs by a diet including omega-3 fatty acids and *Bifidobacterium breve* CECT8242

**DOI:** 10.1007/s00394-023-03165-1

**Published:** 2023-05-06

**Authors:** Gemma Huguet, Irene Puig-Parnau, Jose C. E. Serrano, Meritxell Martin-Gari, María Rodríguez-Palmero, Jose Antonio Moreno-Muñoz, Joan Tibau, Elisabet Kádár

**Affiliations:** 1 Department of Biology, Universitat de Girona, Girona, Spain; 2 IRBLleida-Universitat de Lleida, Avda Rovira Roure 80, 25196 Lleida, Spain; 3 Laboratorios Ordesa S.L., Barcelona Science Park, 08028 Barcelona, Spain; 4 Animal Science-Institut de Recerca i Tecnologia Agroalimentàries, IRTA-Monells, 17121 Monells, Spain

**Keywords:** Pig, Neurogenesis, Neural plasticity, Doublecortin, Arc, Hippocampus

## Abstract

**Purpose:**

Obesity during childhood has become a pandemic disease, mainly caused by a diet rich in sugars and fatty acids. Among other negative effects, these diets can induce cognitive impairment and reduce neuroplasticity. It is well known that omega-3 and probiotics have a beneficial impact on health and cognition, and we have hypothesized that a diet enriched with *Bifidobacterium breve* and omega-3 could potentiate neuroplasticity in prepubertal pigs on a high-fat diet.

**Methods:**

Young female piglets were fed during 10 weeks with: standard diet (T1), high-fat (HF) diet (T2), HF diet including *B. breve* CECT8242 (T3) and HF diet including the probiotic and omega-3 fatty acids (T4). Using hippocampal sections, we analyzed by immunocytochemistry the levels of doublecortin (DCX) to study neurogenesis, and activity-regulated cytoskeleton-associated protein (Arc) as a synaptic plasticity related protein.

**Results:**

No effect of T2 or T3 was observed, whereas T4 increased both DCX+ cells and Arc expression. Therefore, a diet enriched with supplements of *B. breve* and omega-3 increases neurogenesis and synaptic plasticity in prepubertal females on a HF diet from nine weeks of age to sexual maturity. Furthermore, the analysis of serum cholesterol and HDL indicate that neurogenesis was related to lipidic demand in piglets fed with control or HF diets, but the neurogenic effect induced by the T4 diet was exerted by mechanisms independent of this lipidic demand.

**Conclusion:**

Our results show that the T4 dietary treatment is effective in potentiating neural plasticity in the dorsal hippocampus of prepubertal females on a HF diet.

**Supplementary Information:**

The online version contains supplementary material available at 10.1007/s00394-023-03165-1.

## Introduction

Neural plasticity is essential to accommodate new experiences and learn throughout our lives [1]. Once embryonic development of the brain is completed, neurogenesis and synaptic plasticity help to maintain a high degree of environmentally induced neural plasticity. The dentate gyrus of the hippocampus represents one of the adult neurogenic areas which shows synaptic plasticity related to memory and learning processes. Prepubertal age and adolescence mark a crucial period for the maturation of the hippocampus, denoting this stage as a sensitive period during which interventions, such as diets, could have a significant impact on neural plasticity and cognitive function [2].

Different dietary components have been reported to modulate neural plasticity. Excessive intake of certain macronutrients, such as simple carbohydrates and saturated fatty acids, can lead to obesity and attendant metabolic dysfunction. Moreover, excess body weight in midlife has been associated with lower cognitive ability and steeper cognitive decline in late life [3, 4]. Specifically, high-fat (HF) diet has been shown to impair cognitive function both in rodent models and humans [5–8]. Research in rodents has revealed that HF-induced reduction of neurogenesis may be obesity independent, suggesting that dietary fat per se impairs hippocampal neurogenesis [9]. In addition, HF diets reduce the levels of brain-derived neurotrophic factor (BDNF) in both rodents and humans [7, 10]. HF diets also reduce other plasticity-related proteins, such as activity-regulated cytoskeleton-associated protein (Arc) [11], cAMP response element-binding protein (CREB) and synapsin I [8], as well as impair *N*-methyl-d aspartate receptor (NMDAr)-related glutamate transmission [12].

In contrast, other dietary components have been reported to positively modulate neural plasticity. Docosahexaenoic acid (DHA) is an omega-3 polyunsaturated fatty acid that has become increasingly interesting as a dietary component that may potentiate cognitive performance. A greater intake of DHA and eicosapentaenoic acid (EPA) by preschool-aged children has been associated with better cognitive test outcomes [13]. DHA supplementation has also demonstrated to induce slight improvements in learning capacities of children with attention-deficit/hyperactivity disorder [14]. Kawakita et al. [15] proved that dietary DHA supplementation stimulates hippocampal neurogenesis in rats and is an essential molecule for neuronal differentiation. However, other interventional studies in clinical trials with patients with mild cognitive impairment or Alzheimer disease find minor or nonexistent cognitive improvements following the treatment with omega-3 [5, 16], suggesting that its combined use with other dietary factors could enhance DHA effects on cognitive function. For instance, DHA has previously shown synergistic effects with probiotics in regard to metabolic and inflammatory regulation [17, 18]. Interestingly, probiotics are novel nutritional candidates for potentiating and protecting cognition. Different studies have found protective effects against memory deficit in mice, as well as improved learning and memory ability in both aging rats and Alzheimer disease rodent models [19–22]. Recent studies demonstrate that *Bifidobacterium breve* suppresses cognitive deficits and synaptic dysfunction while restoring the levels of BDNF and postsynaptic protein PSD95 in an Alzheimer disease rodent model [23]. Moreover, a study examining the effects of *B. breve* supplementation demonstrated a reduction in cognitive decline in older adults with mild cognitive impairment [24].

Despite the encouraging findings regarding diet effects on hippocampal plasticity shown in rodent models, there is great evolutionary distance between rodents and humans and their translational value is far from perfect. Instead, the pig serves as a good model because of its similarity to human nutrition requirements, comparable gastrointestinal development [25] and similar gyrencephalic brain development [1]. Moreover, similar to humans, the major brain growth spurt in pigs extends from the late prenatal to the postnatal period [1, 26] and their neurodevelopment is also susceptible to dietary factors [27, 28].

The aim of this work was to analyze the effects of two dietary treatments including omega-3 fatty acids and/or probiotic *B. breve* CECT8242 on hippocampal synaptic plasticity and neurogenesis in prepubertal female pigs on a HF diet, and to compare them with the effects of both normal and HF diets. For this purpose, we performed immunolabeling of two widely accepted neural plasticity markers, doublecortin (DCX), a microtubule-associated protein which is expressed in both lineage-restricted neuronal progenitors and immature granule cells of the DG [29], and Arc, a synaptic plasticity-related protein that interacts with specific effector proteins in neuronal compartments and is critical to memory consolidation [30].

## Materials and methods

### Animals and treatments

**Fig. 1:**
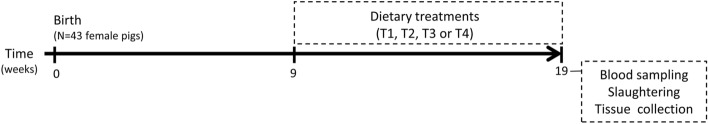
Schematic representation of the experimental design. A total of 43 female piglets turning 9 weeks old were randomly distributed into 4 experimental groups, each consisting in a different dietary treatment. T1: conventional diet (*N* = 11), T2: Western-type diet (high-fat and high-saccharose diet, protein of animal origin) (*N* = 10), T3: western-type diet with protein of vegetal origin and *B. breve* probiotic (*N* = 11), T4: western-type diet with protein of vegetal origin, *B. breve* probiotic and omega-3 fatty acids (*N* = 11). After 10 weeks of feeding, blood samples were collected, pigs were slaughtered and brain samples were obtained, as thoroughly explained in the “Materials and methods” section

A total of 43 female piglets from a Duroc pig line (*Sus scrofa domesticus*) were used in the present study. Animals were born in 11 different litters (i.e., 11 groups of 3–4 littermates). Same sex littermates, from same father and mother, were randomly distributed into 4 experimental groups, using a matched pairs experimental design. After weaning, piglets were transferred to the IRTA pig experimental station, and subjected to the same management procedures as described in Ballester et al. [31] and Jove et al. [32]. Briefly, at 9 weeks of age, animals were located in environmentally monitored facilities, randomly distributed into 4 pens (10–11 animals per pen from different litters) and fed ad libitum for 10 weeks with 4 different dietary treatments giving rise to four different experimental groups: (T1) a conventional (and balanced) growth diet according to Nutrition Resource Centre (NRC) recommendations used as a control diet; (T2) a western-type diet formulated with a high fat and high saccharose content and protein of animal origin (caseinate); (T3) a western-type diet in which 50% of the protein was substituted for protein of vegetal origin (rice hydrolysate) and including 5 × 1010 cfu/day *B. breve* probiotic CECT8242; and T4) a diet similar to T3, supplemented with omega-3 fatty acid (1 g stearidonic acid and 2 g docosahexaenoic acid per 100 g fat) (Fig. 1). Components and nutritional details about the feed provided to piglets have been previously reported in [33] and detailed in supplementary tables S1 and S2, respectively.

Each pen had a partly slatted floor (60% solid concrete and 40% slatted), with some sawdust provided on the concrete floor on a regular basis and a natural light cycle, with a minimum of intensity of 40 lx (EU legislation on pig welfare) and 8 h light. The room temperature was maintained at 22 ± 5 °C.

The experiment lasted until pigs reached 19 weeks of age, when animals were slaughtered after an overnight fasting, at IRTA experimental slaughterhouse in totally controlled conditions and in compliance with all welfare regulations. Pigs were weighed individually at the beginning of the experiment and every two weeks during the whole experiment, as well as on the day before slaughter. Daily food intake (FI; kg/day) was controlled individually by means of automatic electronic feeding system (HOKOFARM, IVO-G^®^, Marknesse, The Netherlands).

### Sample collection

Blood samples for biochemical analyses were taken from overnight-fasted animals immediately before slaughter. After serum separation by centrifugation, lipids and other conventional biochemical variables, including triglycerides (TG) (Spinreact, 1001310), total cholesterol (TC) (Spinreact 1001091), LDL cholesterol (Spinreact 41023), and HDL cholesterol (Spinreact 1001096), were measured using commercial kits from Spinreact (Girona, Spain).

Animals were stunned by exposure to 90% CO_2_ at atmospheric air for 3 min and exsanguinated afterwards. The brain was removed from the skull and dissected to obtain the dorsal part of the left hippocampi, which corresponds to the anterior hippocampus in rodents. The hippocampi were fixed in 4% paraformaldehyde in phosphate buffer saline (PBS), pH 7.4, solution for 4 h and then placed in 15% sucrose in PBS for 3 days at 4 °C followed by 30% sucrose in PBS at 4 °C until they sank. Frozen tissue was cut in a cryostat (Cryocut 1800, with 2020 JUNG microtome) at – 25 °C, to obtain 30 µm sections. Serial sections were collected in six sets, each containing 12 slices, where each slice is 180 µm apart from the next, and stored at – 80 °C until immunohistochemistry staining.

### Immunohistochemistry

Immunohistochemistry labeling protocols were performed by free-floating method. For DCX detection, sections were post-fixed in 2% formaldehyde in PBS for 20 min. Endogenous peroxidase was inactivated by incubation in 0.3% H_2_O_2_ in PBS and the blocking and permeabilization steps consisted of incubation in 0.1% BSA, 0.05% Tween 20 in tris-buffered saline pH 7.6 (TBS) for 30 min plus 30 min in donkey serum 1:200 in Tween 20 in TBS (TBS-T) at room temperature (RT). Sections were incubated in primary antibody anti-DCX (1:600, sc 8066 Santa Cruz) in 0.1% BSA TBS-T, for 1 h at RT and ON at 4 °C. The secondary antibody donkey anti-goat biotin (1:500, Jackson Immunosearch) was applied for 1 h at RT followed by incubation with SA-HRP (1:1800, Perkin Elmer) for 2 h at RT and visualized with diaminobenzidine (DAB) using a DAB substrate kit (Vector, Burlingame, USA). For Arc detection, sections were post-fixed using 3% formaldehyde in PBS for 20 min. After incubation in 0.3% H_2_O_2_ in PBS_,_ tissues were permeabilizated with PBS-T, PBS and PBS-T, and transferred to the blocking solution (0.1% BSA, PBS-T) for 30 min at RT. Sections were then incubated with mouse anti-Arc antibody (1:60 sc 166,461 Santa Cruz, EUA) for 4 h at RT and 72 h at 4 °C. Subsequently, the secondary antibody anti-mouse IgG biotin (1:100, BA-2001, Vector, EUA) was applied for 1 h at RT followed by incubation with SA-HRP (1:3600, Pierce 21124) for 2 h at RT and DAB detection. Finally, sections were mounted onto slides, desiccated at RT 24 h, dehydrated and coverslipped with Pertex mounting medium (Sigma, Aldrich).

### Image acquisition and analysis

Images were obtained using a digital camera (OlympusXC-50) coupled to OlympusVanox-T microscope. Images were analyzed with the Image J v1.50i^*®*^ (Wayne Rasband, National Institutes of Health, EUA) free software.

For DCX analysis, images were obtained using a 20X objective. DCX labeling was measured as number of immunopositive cells/mm using regions of interest (ROIs) along different DG layers. DCX-positive cells were recorded in the crest, the suprapyramidal blade (SP) and the infrapyramidal blade (IP) of the DG. Appropriate gray threshold and particle sizes for DCX cell countings were set for each area and maintained for all subjects. Particle sizes were set to discriminate isolated cells from several overlapping cells, which were manually identified. For Arc analysis, images were obtained using a 4X objective and Arc-positive labeling was recorded in different regions of interest (ROIs) of each hippocampal section: the suprapyramidal blade (SP), the infrapyramidal blade (IP) of the DG, and the hilus, CA1, CA2 and CA3 hippocampal regions. Arc grayscale intensity levels were measured using circular ROIs as area labeled per mm^2^. To remove background noise, each image was digitally smoothed and subtracted from the original. The number of cell countings from DCX or Arc labeling intensity in every region was averaged from three sections from each subject.

### Data analysis

The statistical computer package IBM SPSS Statistics 25.0 was used to process the data. Physiological, biochemical and immunohistological variables were analyzed by univariate one-way ANOVA or Kruskal Wallis test for nonparametric data, except for DCX and Arc immunostaining of DG subregions that were conducted by using GLM repeated measures model. After Levene’s test, post hoc tests were performed for detailed multiple comparisons, using Bonferroni or Tukey HSD if equal variances were assumed (respectively, for univariant or GLM repeat measures) or Games–Howell if they were not assumed. The Spearman’s correlation coefficient rho was used to analyze correlated patterns of DCX and Arc measurements with body weight, TG, TC, LDL, HDL, TC/HDL, along with linear regression line modeling of the main correlations. Significance was considered when *p* value < 0.05.

## Results

### Body weight and serum lipid profiles

All four groups of piglets had similar body weight at the beginning of the experiment (9 weeks old), whereas after dietary treatments, at 19 weeks, the T2 group presented higher body weight (*p* < 0.05) compared to the other three groups, which did not significantly differ between them (Table 1).

**Table 1:** Dietary treatment effect on piglets body weight and serum lipid variables

Group		Mean	SD	*N*	Versus T1 *p* value^1^	Versus T2 *p* value^1^	Versus T3 *p* value^1^
BW 9w kg	T1	16.56	3.08	9			
	T2	16.75	1.96	8	1.000		
	T3	16.14	1.98	11	1.000	1.000	
	T4	16.27	1.37	11	1.000	1.000	1.000
BW 19w kg	T1	53.28	6.38	9			
	T2	61.81	7.75	8	**0.049**		
	T3	51.36	7.03	11	1.000	**0.017**	
	T4	50.23	6.87	11	1.000	**0.006**	1.000
TG mg/dL	T1	20.15	5.02	9			
	T2	29.97	5.56	7	**0.018**		
	T3	33.41	5.87	11	**0.000**	1.000	
	T4	25.15	7.33	10	0.495	0.700	**0.023**
TC mg/dL	T1	128.94	23.58	9			
	T2	163.16	24.17	8	0.152		
	T3	167.16	31.82	11	**0.047**	1.000	
	T4	144.19	36.30	11	1.000	1.000	0497
LDL mg/dL	T1	47.43	8.97	9			
	T2	58.93	7.90	8	**0.050**		
	T3	51.13	9.47	11	1.000	0.345	
	T4	45.33	7.60	11	1.000	**0.010**	0.723
HDL mg/dL	T1	45.22	10.30	9			
	T2	63.17	15.13	7	**0.022**		
	T3	68.91	8.74	11	**0.000**	1.000	
	T4	59.10	11.92	11	0.062	1.000	0.308
TC/HDL mg/dL	T1	2.95	0.79	9			
	T2	2.66	0.71	7	1.000		
	T3	2.46	0.62	11	0.806	1.000	
	T4	2.51	0.74	11	1.000	1.000	1.000

*BW 9w* body weight at 9 weeks old, *BW 19w* body weight at 19 weeks old, *TG* triglycerides, *TC* total cholesterol, *LDL* low-density lipoprotein-cholesterol, *HDL* high-density lipoprotein-cholesterol, *SD* standard deviation

^1^*p* values of GLM multiple comparisons, post hoc Bonferroni, between experimental groups (significant at *p* value < 0.05, highlighted in bold)

Analysis of serum lipid profiles of triglycerides (TG), total cholesterol (TC), high-density lipoprotein (HDL), and low-density lipoprotein (LDL) showed that these parameters were different among the experimental groups, as seen in Table 1. T2 produced increased TG, LDL and HDL levels with respect to control diet T1. In T3, TG, TC and HDL, but not LDL, were also increased with respect to T1. T4 did not show any alteration in the lipid profiles with respect to T1 (Table 1).

### Effects of dietary treatments on hippocampal neurogenesis

**Fig. 2:**
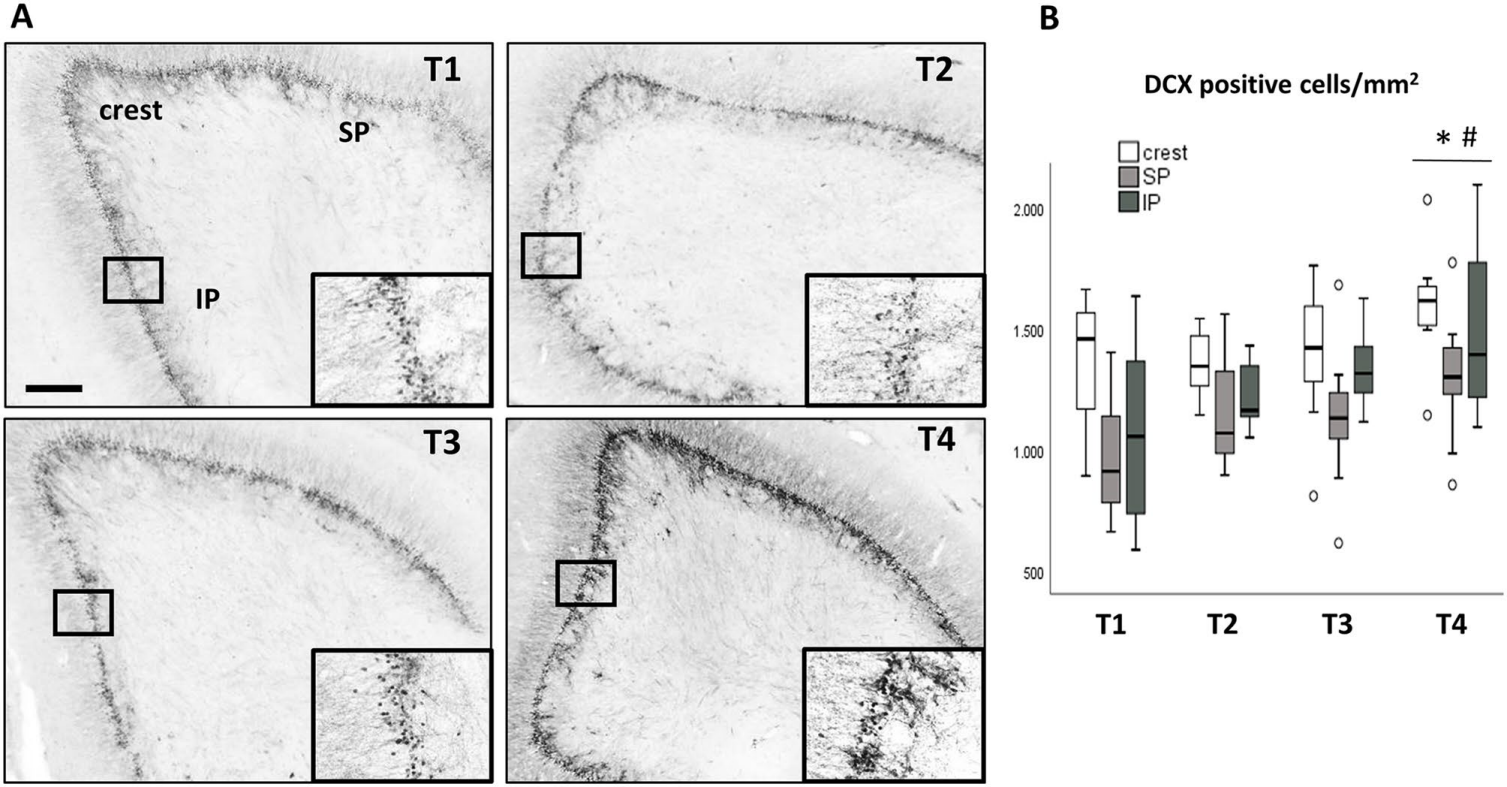
Effects of dietary treatments on DCX-positive cells in DG of hippocampus. **A** Representative photomicrographs of DCX immunopositive cells in the DG of hippocampus in each experimental dietetical group. Scale barr, 400 µm. *SP* suprapyramidal DG blade, *IP* infrapyramidal DG blade. **B** Box plot showing the effect of the different dietary treatments on the number of DCX-positive cells/mm^2^ (± SE) in the crest, SP blade or IP blade of the DG (*p < 0.05 versus T1, ^#^*p* < 0.05 versus T2)

The number of young hippocampal neurons containing DCX was analyzed separately in the dorsal hippocampus. Immunodetection of DCX labeled cells revealed a high level of neurogenesis in the crest and in SP and IP blades of the DG in young female pigs killed at 19 weeks of age in the T4, as shown in Fig. 2.

Dietary treatments caused differences in the number of DCX immunopositive cells in the crest, SP and IP DG regions of hippocampus, analyzed by GLM repeated measures (dietary treatment × DG region). Given that there was no interaction dietary treatment × DG region (*F*_6,58_ = 0.663, *p* = 0.679), the effect of the treatment variable on the three DG regions was analyzed jointly, revealing significant differences between dietetical groups (*F*_(3,29)_ = 4,609, *p* = 0.009) (Fig. 2B). Specifically, the group treated with the T4 presented an increased number of DCX-positive cells compared to the control (T1) and the HF (T2) groups (Tukey HSD, *p* = 0.023 and *p* = 0.022, respectively). No significant differences were observed between T1, T2 and T3 groups.

To determine if hippocampal neurogenesis was related to body weight we analyzed the correlation of total DG DCX^+^ cells and piglet body weight at the time of killing. Although an inverse correlation between body weight and the number of DCX^+^ cells (rho = − 0.413, *p* = 0.010, *N* = 38) in the hippocampus was observed when considering all subjects, this was attributed to differences between groups as it was not observed neither within the control group T1, nor within the other groups. To test if initial body weight influenced subsequent neurogenesis, we also analyzed its correlation with body weight at the onset of the treatments, when piglets were 9 weeks old. No significant correlations were observed between initial body weight and hippocampal DCX^+^ cells at the end of the process, either when considering all the groups together (rho = − 0.284, *p* = 0.084, *N* = 38), or analyzing within each group.

**Fig. 3:**
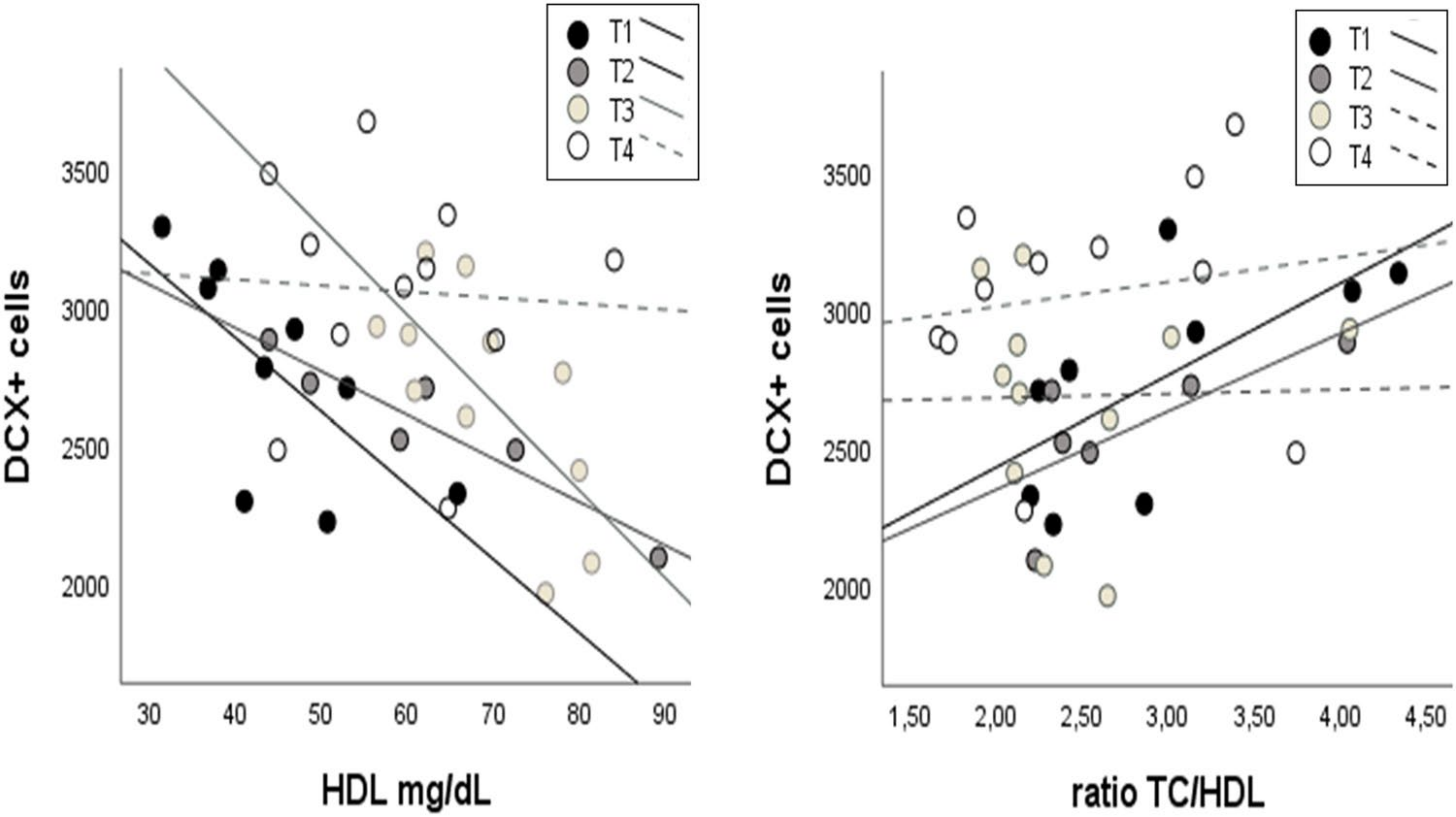
Correlation between DCX+ cells in DG and HDL concentration in serum. Scatter plots showing the relations between DCX+ cells in DG with the concentration of HDL (mg/dL) in serum and with the ratio TC/HDL in the four experimental groups. Regression lines are represented as continuous for significant Spearman correlations (*p* < 0.05), and as spotted in the non-significant correlation, as reported in Table 2

To establish if the changes in hippocampal neurogenesis were related to serum lipid profiles, we analyzed the correlation between total DG DCX^+^ cell number and concentration (mg/dL) of TG, TC, HDL, LDL and TC/HDL. HDL levels presented a negative correlation with the number of DCX^+^ neurons in the T1, T2 and T3 groups, but not in the T4, whereas TC/HDL ratio presented positive correlations in the T1 and T2 but not in T3 and T4 (Table 2, Fig. 3).

**Table 2:** Correlation of total DG DCX^+^ cells and serum lipid variables

	T1	T2	T3	T4
TG mg/dL				
Rho	− 0.517	0.486	− 0.118	0.455
Sig.	0.154	0.329	0.729	0.187
*N*	9	6	11	10
TC mg/dL				
Rho	0.200	− 0.321	− 0.436	0.136
Sig.	0.606	0.482	0.180	0.689
*N*	9	7	11	11
LDL mg/dL				
Rho	0.200	,214	0.436	,136
Sig.	0.606	,645	0.180	,689
*N*	9	7	11	11
HDL mg/dL				
Rho	− 0.717	− 0.943	− 0.682	− 0.45
Sig.	**0.030**	**0.005**	**0.021**	0.67
*N*	9	6	11	11
TC/HDL mg/dL				
Rho	0.717	0.829	− 0.082	0.273
Sig.	**0.030**	**0.042**	0.811	0.417
*N*	9	6	11	11

*TG* triglycerides, *TC* total cholesterol, *HDL* high-density lipoprotein, *LDL* low-density lipoprotein (significant at *p* value < 0.05, highlighted in bold)

### Effects of dietary treatments on hippocampal Arc expression

**Fig. 4:**
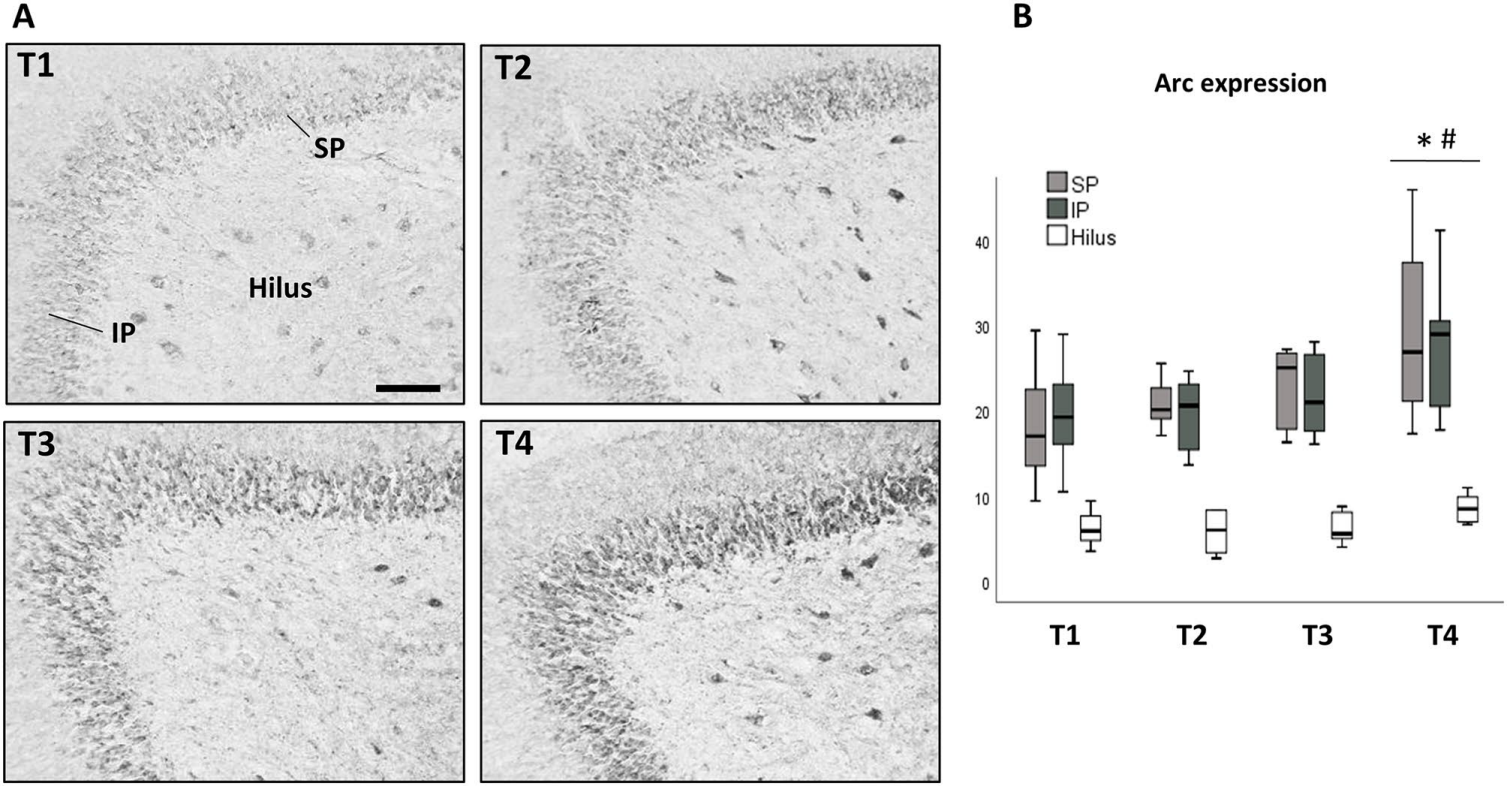
Effects of dietary treatment on Arc expression in the DG. **A** Representative photomicrographs of Arc immunolabeling in the DG from one subject in each experimental dietetical group. Scale barr, 100 µm. *SP* suprapyramidal DG blade, *IP* infrapyramidal DG blade. **B** Box plots show the effect of the different diets on the Arc levels measured as labeled area/ROI (± SE) in the hilus, SP blade or IP blade of the DG (*p < 0.05 versus T1, ^#^*p* < 0.05 versus T2)

Arc immunolabeling in the different subfields of the hippocampus was observed mainly in cell bodies but also in axons and dendrites. Measurement of percentage of area labeled by Arc immunochemistry in the CA1, CA2 or CA3 subfields did not show significant differences in univariate analysis due to dietary treatment (data not shown). The analysis of SP blade, IP blade and hilus DG regions with GLM repeated measures (dietary treatment × region) showed that there was no interaction dietary treatment × region (*F*_6,46_ = 1.382, *p* = 0.242). Therefore, the effect of dietary treatment on Arc expression in the three DG regions was analyzed jointly, showing significant differences (*F*_3,23_ = 4.470, *p* = 0.013) (Fig. 4). Specifically, the T4 group presented increased Arc expression compared to the T1 and T2 groups (Tukey HSD, *p* = 0.021 and *p* = 0.029 respectively). No significant differences were observed between T1, T2 and T3 groups.

No correlations were observed in the different hippocampal subfields between Arc expression and relative body weight nor between Arc expression and the analyzed serum lipid parameters.

## Discussion

Results of the present study show that a diet with high fat content but including the probiotic *B. breve* CECT8242 and omega-3 increased neural plasticity in female prepubertal piglets compared to animals following a conventional HF diet. This diet increased the total number of DCX-positive cells and Arc protein levels in the DG region of the dorsal hippocampus, suggesting a potentiation of neural plasticity in the prepubertal brain. This outcome is especially remarkable since prepubertal age and adolescence mark a crucial period for the development of higher-order cognition through enhanced plasticity of brain circuits [34], and it points to this stage as a sensitive period during which interventions, such as diet, could impact on neural plasticity and cognitive function [2]. Moreover, it has been established that the group at the highest risk of obesity during adulthood are children and young people of prepubertal age with overweight [35], and that attendant metabolic dysfunction has important implications for pubertal development in girls [36]. In light of this, strategies that could influence neural plasticity aimed at normalizing body weight and improving dietary habits before the onset of puberty, gain relevance.

Considering that high-fat diets have been reported to induce alteration of neurogenesis [9, 35] and neural plasticity [7, 8, 10], our initial hypothesis was that administration of the HF would cause a decrease in DCX-positive cells and Arc levels in the prepubertal pigs. Our work is the first study analyzing hippocampal protein levels of Arc in pigs, and to our knowledge, there is only one other study examining the effect of HF diets on DCX cells in pigs. However, in the aforementioned study, the HF diet was administered to sows during gestation and lactation, causing reduction of hippocampal DCX cells in their piglets [27]. Regarding the molecular relationships between DCX and lipid demand, it has recently been show that lipid droplets are highly abundant in adult mouse neural stem/progenitor cells (NSCP), which is significant given that lipid droplet availability could affect NSCP proliferation [38]. On the other hand, it has been demonstrated that the 27-hydroxycholesterol, a circulating cholesterol derivative that can cross the brain–blood barrier, down-regulates hippocampal Arc expression in mice with dietary cholesterol [11]. Additionally, changes in the lipid composition of the diet could have an impact on Arc function since membrane budding events, such as endocytic internalization of AMPA receptors interacting with “lipid rafts”, could be sensitive to differences in the lipidic membrane constituents [39]. Nevertheless, the HF diet used in this study did not lead to quantitative changes in both the analyzed neural plasticity markers in the DG of female piglets. Our results lend support to the assertion that there is an absence of an established metabolic syndrome in these animals at 19 weeks of age, as reported in recently published reports of Jove et al. [32], Ballester et al. [31] and Valent et al. [33]. Their findings integrate with the present results into a multidisciplinary study analyzing the effect of the four dietary treatments and show that the administration of this HF diet caused some anomalies that could trigger the emergence of the metabolic syndrome. The results of these studies showed changes in the circulating and tissular lipidome, transcriptomic changes in adipose tissue related with proinflammatory pathways, upregulation of obesity-related pathways as well as the alteration of appetite-regulatory neuropeptides and neurotransmitters due to the HF diet, which were partially reversed by supplementation with the proposed nutrients [31, 33].

We cannot rule out the possibility that the absence of negative effects of the HF diet on neurogenesis and synaptic plasticity could also be due to a higher availability of nutrients, required in this high-plasticity stage. In this regard, hippocampal neurogenesis had been analyzed in adult macaques showing positive correlations of number of DCX-positive cells in DG with TC/HDL ratio in serum, suggesting that increased neurogenesis is associated with elevated metabolic activity [40]. In support of this hypothesis, we also observed a positive correlation between neurogenesis and lipid ratio TC/HDL, both in T1 and T2 groups. Moreover, we observed a negative correlation between HDL serum levels and the number of hippocampal young neurons for all groups, except for T4 group. Since HDL participates in reverse cholesterol transport from peripheral cells to the liver for further catabolism and excretion [41], these results reinforce the hypothesis that neurogenesis was related to higher lipidic demands. Importantly, inadequate or dysfunctional brain-derived HDL are also implicated in cerebrovascular dysfunctions, neurodegeneration, or neurovascular instability, as shown in a recent review [42]. Moreover, although both HF and cholesterol enriched diets have been shown to reduce Arc expression [11] in rodents, it has also been reported that HF diets increase Arc expression in mice in the early stages of obesity [43], suggesting the involvement of compensatory mechanisms in the response of neural plasticity to HF diet.

Interestingly, T4 exerted a positive effect on neurogenesis. The increase in DCX-positive hippocampal cells was independent of the TC/HDL lipid ratio or of the HDL levels, suggesting that the bioactive ingredients triggered alternative mechanisms to induce this neurogenic effect. Thus, despite the growing evidence in support of nutritional lipids playing an important role of in the regulation of neural stem cell niche, the mechanism of action nor the critical exposure time are clear [44] and further studies are required to establish the exact relationships between dietary lipids and adult neurogenesis.

On the other hand, the DG protein Arc levels were also found increased in T4 experimental group, despite no correlations being observed between Arc levels and the physiological parameters. This neural plasticity marker has been associated with the improvement of HF diet-induced cognitive impairment in mice. Deficiency of Cyp1b1 has been found to protect the animals from HF diet-induced learning and memory deficits, and has also been shown to increase Arc expression [45]. Considering this relation to cognitive improvement, it is particulary interesting that the T4 dietary treatment increased DG protein levels of Arc in pigs, including in a HF dietary context.

Therefore, this study shows that dietary supplementation with bioactive T4 compounds not only reverses HF-induced lipidomic and appetite-related neurological abnormalities observed in the previous studies [31, 33], but can also enhance neuronal plasticity.

These analyses were performed in the dorsal hippocampus, but a previous analysis of the ventral hippocampus did not detect any changes in neurogenesis (data not shown). This suggest that neurogenic potentiation by this dietary supplementation was specific for the dorsal hippocampus (denominated anterior in rodents), an area that plays a preferential role in cognition and memory, whereas the posterior (ventral) hippocampus regulates emotion [29].

It has been reported that supplementation of preterm pigs with *B. breve*, plus fructo-oligosaccharide and glutamine, improved cognitive performance [28]. Our results show that T3 diet, including the probiotic, did not increase the number of DCX-positive cells nor Arc protein levels in DG. However, it did alter the correlation of lipid TC/HDL ratio and the neurogenesis marker. Interestingly, Bernier et al. recently demonstrated that niacin production by *B. breve* could protect against lipid droplet alterations and neuroinflammation [46]. As mentioned above, lipid droplet availability could affect NPSC proliferation [38]. Considering that T4 diet only differs from T3 in the addition of omega-3 fatty acids, the neurogenic and plasticity effects of T4 diet could be attributed to the DHA. Despite DHA having been previously shown to enhance neurogenesis [15, 37, 47, 48] and regulate the expression of neuronal plasticity markers including Arc [49, 50], synergistic effects with the other components of the diet such as *B. breve* CECT8242 could not be discarded. In this regard, it has been reported that either omega-3 fatty acid or a mixture of probiotics is able to regulate lipidic profile, insulin sensitivity and inflammatory markers, but it was the addition of the two components together which had more pronounced effects [17, 18]. One limitation of our study is that it was not designed to analyze the effect of the individual components of the formulated diets, and so their synergics could not be explored. As far as we know, the synergistic effects of omega-3 fatty acids and probiotics on neural plasticity or on cognition have not been studied yet, and our results particularly encourage further exploration of whether DHA and the probiotic *B. breve* CECT8242 act synergistically to potentiate these processes.

## Conclusion

The present study shows for the first time that the T4 diet, including the *Bifidobacterium breve* CECT8242 and omega-3 fatty acids bioactive supplements, potentiates neural plasticity in the dorsal hippocampus of prepubertal brain of females fed with a high-fat diet. In addition, our findings show that the number of DCX-positive cells but not Arc protein levels, in the DG of piglet prepubertal brain, positively correlates with high cholesterol demand, in animals subjected to both control and HF dietary treatments. Furthermore, we observe that the neurogenic effect induced by the dietary treatment with the two bioactive supplements is exerted by mechanisms independent of this lipidic demand. Considering that HF diets can have negative neuroplasticity effects, these are promising results as they show that this dietary treatment could be effective in potentiating neuroplasticity during brain development, even in the context of a HF diet consumption.

## Supplementary Information

Below is the link to the electronic supplementary material.

Supplementary file1 (XLSX 12 KB)

Supplementary file2 (XLSX 11 KB)

## Data Availability

Data analyzed in this study are contained within the article or supplementary material file. Remaining data that support the findings of this study are available from the corresponding author upon reasonable request.
